# Cancer/testis antigens and gametogenesis: a review and "brain-storming" session

**DOI:** 10.1186/1475-2867-5-4

**Published:** 2005-02-16

**Authors:** Martins Kalejs, Jekaterina Erenpreisa

**Affiliations:** 1 Biomedical Research and Study Centre of the Latvian University, Riga, Latvia

**Keywords:** CTA genes, gametogenesis, polyploidy

## Abstract

Genes expressed both in normal testis and in malignancies (Cancer/ Testis associated genes – CTA) have become the most extensively studied antigen group in the field of tumour immunology. Despite this, many fundamentally important questions remain unanswered: what is the connection between germ-cell specific genes and tumours? Is the expression of these genes yet another proof for the importance of genome destabilisation in the process of tumorigenesis?, or maybe activation of these genes is not quite random but instead related to some programme giving tumours a survival advantage?

This review collates most of the recent information available about CTAs expression, function, and regulation. The data suggests a programme related to ontogenesis, mostly to gametogenesis. In the "brain-storming" part, facts in conflict with the hypothesis of random CTA gene activation are discussed. We propose a programme borrowed from organisms phylogenetically much older than humans, which existed before the differentiation of sexes. It is a programme that has served as a life cycle with prominent ploidy changes, and from which, as we know, the germ-cell ploidy cycle – meiosis – has evolved. Further work may show whether this hypothesis can lead to a novel anti-tumour strategy.

## Introduction

Cancer/Testis (CT) antigens are a group of tumour antigens with gene expression restricted to male germ cells in the testis and to various malignancies. Their function in tumours is enigmatic and a common between testis genes (gametogenesis) and cancer remains elusive. When the causal link is not evident, it is tempting to believe the association is random, and assign it to general aspects of "genome instability in cancer". However, we believe a more direct link may exist. As suggested in this review, possible clues may be found in the common evolutionary pathway between ploidy cycles in meiosis and polyploidy in tumour cells. The latter, along with CT antigen expression, is a characteristic feature of well-progressed tumours. However, before discussing such possibilities, it is necessary to review the established literature.

The search for tumour antigens began in the 1960's with two groups identifying first alpha-fetoprotein (AFP), a serum marker for hepatoma and germ-cell tumours [1], and then carcinoembryonic antigen (CEA), a serum marker for colon and other epithelial cancers [2]. These antigens were discovered using heterogenous sera acquired by immunizing laboratory animals with human tumour material. However, only during the 90's did both cellular [3,4] and humoral [5] immune responses to human tumours get proper molecular definitions.

The first CTA, MAGEA-1, was identified in 1991 by Boon and colleagues using T-cell epitope cloning, a very complicated and time-consuming method [3]. In 1995 the SEREX (serological expression cloning) technique to identify tumour antigens was developed by Pfreundschuh and colleagues [5], which remains the leading approach to identifying new antigens that elicit humoral immune responses. Besides MAGEA1, BAGE, and GAGE1 discovered by T-cell epitope cloning, SEREX very soon displayed more tumour antigens with a cancer/testis restricted expression profile (SSX2, NY-ESO-1, and SYCP-1). The term "cancer-testis (CT) antigen" was introduced by Chen et al. [6], who recognized this group of genes had little in common except their expression profile.

By initial definition, expression of genes coding for CT antigens should be restricted in normal tissues to male germ cells in the testis and to malignancies of various histological types. However, the criteria proposed in the 90's are not true for all antigens of this group as seen today. Furthermore, for many of the recently discovered gene products with the described expression profile, no T-cell recognized epitopes have hitherto been identified. This is why CTA – "Cancer/Testis Associated" is a more appropriate name for this family of genes (and will be used in this context further in this review), because a lot of its members still need to be proven as possessing antigenic properties in cancer patients.

Attributing genes to the CTA gene family is based on several characteristic features [7,8]:

1. Predominant expression in germ cells of the testis and generally not in other normal tissues.

2. Expression in a number of malignant tumours of various histological types.

3. Mapping of the gene to the X-chromosome

4. Membership of a multigene family.

5. Antigenic properties in tumour-bearing patients.

Some exceptions to these criteria for certain CTAs will be described and discussed later.

### Expression of CTA genes

To date, 89 individual CTA genes or isoforms have been described, which are organised in to 44 families (see additional file 1). From these, 19 families are testis-restricted, and 11 show additional expression in one or two somatic tissues. Nine are expressed in 3–6 tissue types besides testis, and 5 are ubiquitously somatically expressed. With the exception of the testis-restricted CTAs, the others also show expression in the pancreas but at levels as much as 10 × lower than in testis (based on mRNA expression levels from [9]). Expression of CTA was first shown in melanoma and all the classic CTA are expressed in this type of tumour, but since the 1980's, expression in various other tumours has been recognised (additional file 2).

The expression pattern of CTAs during spermatogenesis is of special interest. Functional analysis of these genes during gametogenesis might well give some clues about their possible role in tumours. Their expression is restricted exclusively to spermatogenic germ cells with other tubular cells (e.g. Leydig and Sertoli) being negative. This fits well with the findings of Yuasa et al. [10] who demonstrated that CTAs have much higher expression frequencies in the germ cell cancers (seminomas) than non-seminomas..

**Figure 1:**
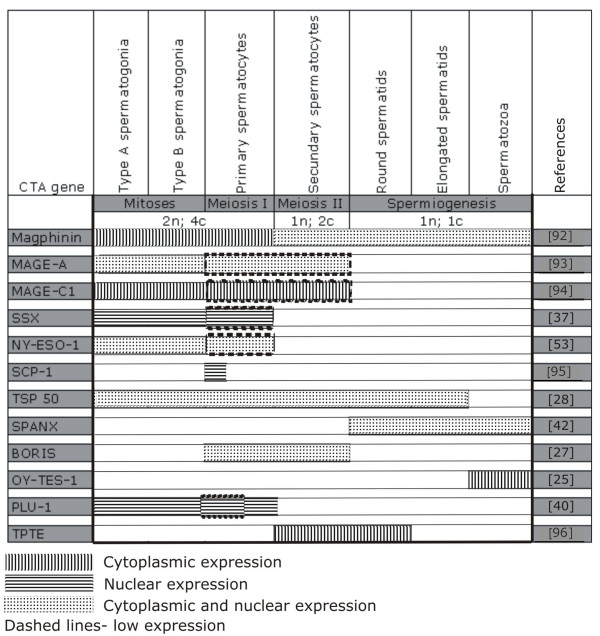
Expression of CTAs during male germ cell development. References for Fig. 1: [25] [27] [28] [37] [40] [42] [53] [92] [93] [94] [95] [96]

Different CTAs are expressed during different stages of spermatogenesis (Fig. 1.), so one may imagine that their functions are versatile, starting from regulation of mitotic cycling in spermatogonia, association with the meiotic cycle in spermatocytes, and finalizing with acrosome maturation in sperm.

In normal tissues, expression of NY-ESO1, MAGE-A3, -A4, and -A8 through -A11 as well as of several members of the XAGE gene family is found in the placenta. NY-ESO1 and several XAGEs are also expressed in the fetal ovary [11-13].

### Regulation of CTA expression

The mechanisms involved in regulation of CTA expression have recently been comprehensively reviewed by Albert Zendman et al [14]. Thus, only a short recitation of some of the main points is provided here.

Mostly, methylation processes are responsible for the ectopic derepression of CTA genes. Using the demethylating agent, 5-aza-2-deoxycytidine (5DC), expression of several CTAs in cultured tumour cell lines can be induced/upregulated [15]. 5DC entraps DNA methyltransferases in a complex with DNA, which leads to progressive loss of DNA methylation, thereby releasing transcriptional blockage. Such upregulated expression has been reported for several MAGE members – LAGE-1, SSX-2, CAGE, NY-ESO-1. For MAGE-A1, demethylation is necessary and sufficient for gene expression, suggesting demethylation is the primary mechanism of transcription control [16]. In this context, a recent discovery of a CTA, *viz*. 'Boris', is interesting. Boris is reported as being expressed in several types of malignancies, and normally plays a major role in regulating methylation processes during spermatogenesis – it removes imprinting from genes during the last mitotic division of spermatocytes (reviewed in [17]). Several lines of evidence indicate that expression of some CTAs is dependent not only on demethylation, but on other transcriptional mechanisms.

Histone deacetylase (HDAC) inhibitors, on their own or in combination with 5DC, can also induce CTA expression, including MAGE, SSX, and NY-ESO-1 family members [15]. The CTA-rich region in Xp11.21-22 (e.g. SSX, MAGE-B) may escape X-chromosomal inactivation, but these genes are not normally expressed in females [18]. While global hypomethylation is common and prominent in colorectal cancer, few CTAs have ever been reported as expressed in this type of cancer [19]. Non-demethylation dependent induction of MAGE expression has been demonstrated by Park et al. [20], demonstrating that 40 mM NaCl induces the transcriptional and translational activation of MAGE-B1 and -B2 in specific tissues at hypertonic conditions.

There exist definite expression patterns (sets) of different CTAs in certain tumours. Marked heterogeneity of CTA expression is found in cells of some tumours, which cannot easily be explained by a global demethylation process [21-24]. The mechanisms of ectopic transcriptional activation of CTA genes clearly needs more investigation.

### Function

Information regarding the function and cellular localization of CTAs is far less comprehensive. Often the proposed function is based purely on sequence homology with another protein of a known function. The only CTA proteins functionally established in gametogenesis are SCP-1, involved in chromosome pairing during meiosis [7], OY-TES-1 which functions in acrosin packaging in the acrosome of sperm heads [25], SPO11 acting as a meiosis-specific endonuclease [26], and BORIS, which is involved in cancellation of imprinting by epigenetic reprogramming during the final round of mitosis in spermatogenesis [27]. However, contrary to the situation in meiosis where it is rapidly degraded after the meiotic prophase in spermatocytes, SCP-1 expression in tumours is not cell cycle restricted [7]. BORIS is a paralog of CTCF. CTCF is a highly versatile 11 zinc-finger factor involved in various aspects of gene regulation – X chromosome inactivation, reading of imprinting sites, etc. [27]. During spermatogenesis, Boris is expressed later than many other CTA genes ([27]; Fig. 1.).

Suggestions for the functions of other CTAs mostly arose from studies of their homology with some well-known proteins and their domains, TSP50 being protease-like, CT17 phospholipase-like, and CT15 metalloproteinase-like [28]. The CT15 gene encodes a disintegrin and metalloproteinase (ADAM) domain 2, which is a member of the ADAM protein family [29]. Members of this family are membrane-anchored proteins structurally related to snake venom disintegrins, which have been implicated in a variety of biologic processes involving cell-cell and cell-matrix interactions, including fertilization, muscle development, and neurogenesis. This member is a subunit of an integral sperm membrane glycoprotein called fertilin, which plays an important role in sperm-egg interactions [30]. It is a membrane metalloproteinase with a possible role in tumour evasion and metastasis.

LDHC, the germ cell-specific member of the lactate dehydrogenase family, escapes from transcriptional repression, resulting in significant expression levels in virtually all tumour types tested. It might contribute to the constitutive activation of an anaerobic pathway in tumours, because its expression in tumours is not dependent on hypoxia [31].

Suggestions as to the role of MAGEs with a CTA expression profile mainly depend on studies of their ubiquitously expressed family members (e.g. Necdin, MAGE-D1, NRAGE, Dlxin-1). In general, the data point to a role for MAGEs via transcriptional regulation in cell cycle control and apoptosis. F.ex, Necdin-related MAGE proteins differentially interact with the E2F1 transcription factor and the p75 neurotrophin receptor [32]. The high level of homology among members of the MAGE family in both mouse and human suggests an important function both in testis and cancer.

The products of the SSX genes belong to the family of highly homologous synovial sarcoma X (SSX) breakpoint proteins. These proteins may function as transcriptional repressors; SSX1, SSX2 and SSX4 genes have been involved in the t (X;18) translocation characteristically found in all synovial sarcomas [33-35]. This translocation results in the fusion of the synovial sarcoma translocation gene (SYT) on chromosome 18 to one of the SSX genes on chromosome X. The encoded hybrid proteins are probably responsible for transforming activity. In the nucleus of sarcoma cells, both diffuse and speckled localisations of SSX protein have been reported [36,37].

**Figure 2:**
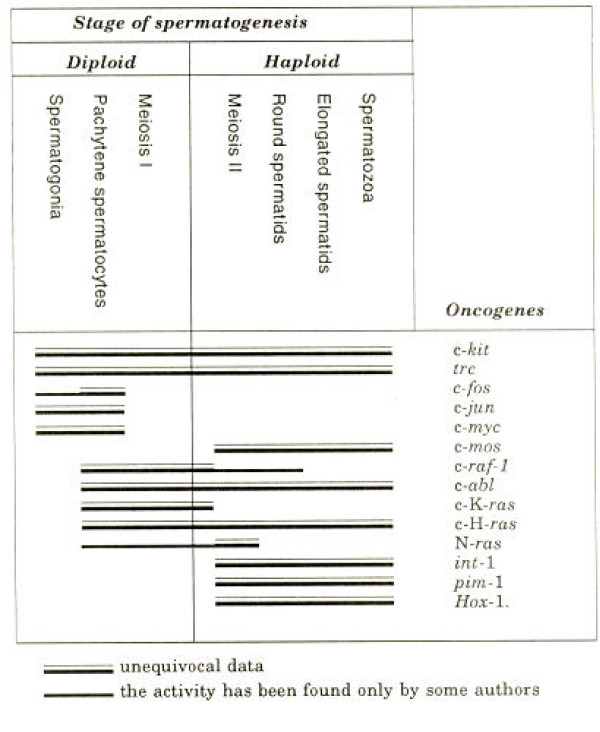
Oncogene expression during spermatogenesis (mainly in mice). From [70].

The HOM-TES-85 protein has structural peculiarities that are shared exclusively with the N-myc oncoprotein. However, functional studies are required for confirmation [38]. Besides, the C-myc proto-oncogene is a normal participant of spermatogenesis (Fig. 2).

BRDT is similar to the RING3 protein family. It possesses 2 bromodomain motifs and a PEST sequence (a proline, glutamic acid, serine, and threonine cluster) characteristic of proteins that undergo rapid intracellular degradation). The bromodomain is found in proteins that regulate transcription [39].

PLU-1, a large multi-domain nuclear protein also has a strong transcriptional repression activity. It is a member of the ARID family of DNA-binding proteins. Plu-1 mRNA and PLU-1 protein are both highly expressed in the mitotic spermatogonia. The expression is reduced in the early prophase I stages (leptotene, zygotene), but reappears at pachytene, still being detectable in diplotene cells. It is located diffusely over the nucleus. PLU-1 might have a role in regulating meiotic transcription, restricted to certain meiotic stages [40].

The protein encoded by the SPANX gene targets the nucleus and associates with nuclear vacuoles and the redundant nuclear envelope in sperm cells [41]. *In situ *hybridization of human testis sections showed SPAN-X mRNA expression in round and elongated spermatids [42]. These redundant nuclear envelopes have a unique structure of limited chromatin sheets continued as annulate lamellae. Both these enigmatic structures have also been described in intact lymphomas [43] and irradiated lymphomas [44].

The protein encoded by IL13RA1 gene is a subunit of the interleukin-13 receptor. This subunit forms a receptor complex with IL-4 receptor alpha, a subunit shared by IL-13 and IL-4 receptors. This subunit serves as a primary IL-13-binding subunit of the IL-13 receptor, and may also be a component of IL-4 receptors. This protein binds tyrosine kinase TYK2, and thus may mediate the signalling processes that lead to the activation of JAK1, STAT3 and STAT6 induced by IL-13 and IL-4 [45].

SGY-1 (soggy-1), a secreted protein related to the Dickkopf protein family, is involved in suppressing the Wnt signal-transduction pathway controlling transcription activation of genes such as c-myc, c-jun, Fra, and cyclin D1 by preventing the accumulation of beta-catenin [46,47]. Wnt proteins are implicated in a wide variety of biologic processes including cell fate determination and patterning in early embryos, and in cell growth and/or differentiation in certain adult mammalian tissues [48]. Wnts can induce proliferation in different types of stem cells [49,50]. The importance of Wnt signalling during tumorigenesis has been recently emphasised [51,52].

NY-ESO-1 is one of the most immunogenic and therapeutically promising CTAs but functional studies on this gene are severely lagging behind its practical application. Unlike the majority of CTA genes, NY-ESO-1 stops its expression in well-progressed tumours, so it can be used as a marker to follow the early progression of testicular tumorigenesis [53].

CAGE [54] and HAGE [55] code for proteins with helicase-like features. Probably, it might be involved in recombination exchange in testis and recombination DNA repair in tumours.

TPX-1 is now seen as an integral protein of the outer dense fibres and the acrosome of spermatids in rats [56].

In summary, we see that the functions of individual CTA, when known, are very diverse, including, for example, both activators and repressors of proliferation and transcription.

### Immunogenicity

Immunogenicity in cancer patients is elicited only by short peptide sequences of CTA epitopes, which are presented on the tumour cell surface by HLA Class I molecules in the case of cytotoxic T lymphocyte (CTL) mediated immune responses and by HLA Class II molecules on the surface of APC (antigen presenting cells), in the case of T-helper cell (T_H_) mediated immune responses. Identification of these epitopes is one of the main goals of CTA research. Knowledge of epitopes recognized by the immune system allows the creation of the tumour-specific vaccines. In the vaccination process, T-cell epitopes are often administered together with different adjuvants or cytokines, or delivered using peptide pulsed autologous dendritic cells, all of which are aimed at enhancing the immune reaction [57]. Currently efforts are being made to identify HLA class II restricted epitopes in order to promote T_H _responses, which are required to support the activity of CTLs, and provide a more "complete" immune response. However, vaccines designated to prime the immune system against tumours expressing various CTA have so far shown only partial clinical success [57-59].

Since the technique used to identify candidate tumour antigens has changed from T-cell epitope cloning [3,60], and SEREX (serological analysis of cDNA expression libraries) [5,61,62] to differential gene expression analysis by various techniques like RDA (representative difference analysis) [63], DD (differential display) [64] and SSH (suppression subtractive hybridization) [65] – and even further to bioinformatics – we cannot really be sure about the adequate use of the definition "antigen" in relation to CTA gene products. Whilst the former two techniques are dependent on the immunogenicity of specific epitopes in cancer patients, the latter ones are simply based on mRNA expression levels or even sequence homologies detected via search engines and provide no answer about immunoreactivity. In additional file 1, one can see that an immune response is documented against only 19 of 44 CTAs. Immune recognition of the majority of these CTAs is cancer-related, occurring spontaneously in cancer patients, not in cancer-free individuals. The exceptions to this are humoral immune responses to MAGEB1/CT3.1 in systemic lupus erythematosus patients [66] and to SPA17/CT22 in vasectomised men [67].

CTA can be considered to be tumour specific. There exists the blood-testis barrier [68], which prevents the immune system from contacting with CTA gene products. Besides this, germinative cells do not express HLA class Ia molecules [69], so they cannot present their expressed proteins to the immune system. For these reasons, the immune system never comes into contact with these proteins and recognizes them as "non-self" structures.

### Brain-storming

The function of most CTAs is unknown, although some role in regulation of gene expression (both activating and repressing) seems likely [8]. Are CTAs oncogenes? By definition, oncogenes are normal cellular genes participating in proliferation cascades, which are abnormally activated in tumours [70]. Therefore, with the exception of one (HOM-TES-85, which has structural homology with the myc-oncogene) CTAs can not formally be regarded as oncogenes. Are CTAs simply activated by the imbalanced genome, due to its instability in tumours? Some, such as SSX, whose ectopic expression is caused by the SYT-SSX fusion due to translocation t (X;18) in synovial sarcomas, certainly are. However, a large number of CTA genes are only located on the X-chromosome and this chromosome is not a specific site of chromosome breaks and translocations usually associated with tumours [71]. However, the chromosome region 20q13.2 containing the BORIS gene is commonly amplified or exhibits moderate gains of material in many human cancers. This has strengthened the idea that this region contains a major oncogene [72,73]. A preliminary report using RT-PCR has found that BORIS expression is detectable in over half of ~200 cancer cell lines studied, representing most of the major forms of human tumours, discussed in [17]. However, this observation awaits further confirmation. Theoretically, the aberrant expression of the amplified (by chance) Boris, which by analogy with CTCF may demethylate CTA genes located on X-chromosome, may in turn activate a large body of CTA genes in human malignancies. However, why are other gametogenesis-related CTA genes also activated from other chromosomes? It does not look like a chance event and therefore amplification of "Boris" may still not be random.

Diversity of functions, including genes involved in ontogenesis suggest that CTAs are activated either as a result of the genome instability (however, why testicular genes?) or as part of a complex program. Also from this point, this looks like a program related to gametogenesis. The same idea was proposed by Old [8].

If CTAs generally do not enhance tumour growth except by stimulating proliferation, like oncogenes, another possibility is that they could do it by stimulating DNA repair or inhibiting apoptosis (combination of all three is possible). Indeed, some CTAs have relation to the DNA repair factors by homologous recombination. These are SPO11, SYCP1, helicase-like CAGE and HAGE acting in meiotic prophase of gametogenesis. In turn, homologous recombination in tumours was shown to act anti-apoptotically [74].

Are these CTAs restricted only to prophase of meiosis, where recombination takes place? It appears not. For example, some are associated with the spermatogonial stage (Plu-1 mRNA and PLU-1), and some with maturation of the acrosome (see Fig. 1). It is a program again, and why only male gametogenesis? But it also occurs in oogenesis [8]. Thus, prophase of meiosis (pairing and recombination) may be still the most important.

So, ectopic activation of CTA genes is not entirely random, being induced from the sexual X-chromosome but also from loci on other chromosomes, relating to gametogenesis. However, it is curious then that the very common proto-oncogenes of proliferatives cascades also participate in gametogenesis, e.g. myc, ras, jun, etc. This can be seen in Fig 2, (taken from the Janis Erenpreiss [70]). He and others postulated a link between gametogenesis and cancerogenesis before CTAs were revealed [75-77].

In turn, Old [8] looked at the problem from a new angle and suggested that CTAs provide a causal link between gametogenesis and cancer. This seems plausable, but does this illegitimate program in tumours embrace, even mosaically, only gametogenesis? May be only the DNA recombination repair component is common? But the CTA genes are repressing a wide Wnt family, which also functions in early embryogenesis.

The program sounds more like an ontogenetic (life-cycle) one. Let us remember the experiments by Mintz and Illmeisee [19] who cloned normal genetic mosaic mice by introducting the nuclei of malignant teratocarcinoma into enucleated eggs. Both gametic, parthenogenetic and trophoblastic theories of cancer have also been proposed in the past [8,70] and can be viewed as embryonal or ontogenetic theories of cancer.

Another question is whether CTA genes govern a life-cycle-like program with its key events similar to meiosis? In turn, to which process does meiosis provide a key – recombination and reduction division? Are tumours capable of these? We also have to consider why X-chromosomes are involved, and not Y-chromosomes, which are responsible for sex? Perhaps this is because the X-chromosome is better conserved evolutionary and appeared prior to sex discrimination.

So, this looks like an ontogenetic program, which evolutionary preceded sexual (amphimictic) life-cycle, with the key in recombination and reduction division; but what is it? The answer is evident – ancient ploidy cycles [78,79]. In general, ploidy cycles are displayed as a cyclic increase and reduction of ploidy (chromosome number), and these involve the pairing of homologous chromosomes and their segregation, omitting one round of DNA replication.

There may be reduction of ploidy 2n-n (sexual meiosis) or from nx to 2n DNA numbers, in the asexual ploidy cycles characteristic for a few protists, as in Amoebae and foraminiferans [80,81] as well as part of ontogenetic programs in the more developed taxons [82,83]. Contrary to gametic reduction in sexual meiosis, the reduction of ploidy in polyploid somatic cells is called "somatic reduction".

In this context, expression of some CTAs by the placenta is of interest because trophoblast and decidua are the only mammalian tissue capable of endoreduplication creating enormously large ploidies [84]. an ability shared by many tumour cells. The latest studies on a silver fox revealed somatic reduction in the giant cells of a trophoblast [85].

Now let us return to DNA recombination repair in tumours as a means of survival, as already mentioned. Repair by homologous recombination can protect malignant tumour cells from apoptosis. In particular, as shown in our laboratory, endopolyploid cells employ this mechanism [86]. Likewise, expression of CTAs – endopolyploidy – is a hallmark of malignant tumour progression where there is deficient TP53 function [87].

**Figure 3:**
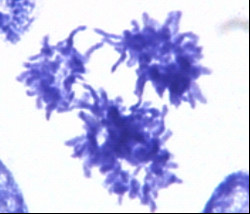
Reductional mitotic divisions generating low ploidy cells from one large polyploid cell. Non-treated Burkitt's lymphoma cell line, DNA staining with Toluidine-blue. × 2,500.

In turn, some giant tumour cells show the capability to segregate their genomes and return to mitosis ([86,88-91], see Fig. 3).

## Conclusion

A hypothesis is put forward that activation of at least some of the CTA genes in p53-deficient human tumours could be due to the genetic program running "relic" ploidy cycles in tumour cells. This hypothesis offers new opportunities for the design of novel tumour treatment strategies. In particular, passive therapy using CTAs to prime the host immune system against the tumour could be replaced with gene therapy aimed to block the function of CTA gene products or even their expression. This approach is promising, because normally only the germ cells in the testis express CTA genes and they are well protected by the blood-testis barrier. Thus, there should not be any problem with tumour-specific priming, and respectively we would predict there to be no side effects.

## Supplementary Material

Additional File 1 "Chromosomal localization, type of immune response, identification method and identification references for all known 44 CTA gene families". Click here for file

Additional File 2 " CTA Frequency (%) of expression in various tumour types". Click here for file
