# Supplementary material for: Cancer/testis antigens and gametogenesis: a review and "brain-storming" session
Source: Cancer Cell Int. 2005 Feb 16;5:4. doi: 10.1186/1475-2867-5-4 (PMC552320; doi:10.1186/1475-2867-5-4)
Supplement: Additional File 1 — "Chromosomal localization, type of immune response, identification method and identification references for all known 44 CTA gene families". [file 1475-2867-5-4-S1.doc]

**Additional file 1.**

**Chromosomal localization, type of immune response, identification method and identification references for all known 44 CTA gene families.**

| **CT GENE  FAMILY** | **CT IDENTIFIER**** | **LOCUS** | **FAMILY  MEMBERS** | **IMMUNE  RESPONSE** | **ID METHOD** | **REFFERENCES** |
| --- | --- | --- | --- | --- | --- | --- |
| MAGEA | CT1 | Xq28 | 12 | cellular and humoral | T cell epitope cloning | [1] |
| BAGE | CT2 | 13, 21 | 5 | cellular | T cell epitope cloning | [2] |
| MAGEB | CT3 | Xp22-p21 | 5 | cellular and humoral | Positional cloning | [3] |
| GAGE | CT4 | Xp11.4-p11.2 | 8 | cellular | T cell epitope cloning | [4] |
| SSX | CT5 | Xp11.23-p11.22 | 4 | cellular and humoral | SEREX | [5] |
| NY-ESO-1 | CT6 | Xq28 | 3 | cellular and humoral | SEREX | [6] |
| MAGEC | CT7 | Xq26 | 2 | humoral | SEREX, RDA | [7] |
| SYCP1 | CT8 | 1p13-p12 | 1 | humoral | SEREX | [8] |
| BRDT | CT9 | 1p22.1 | 1 | N/A | Database mining | [9] |
| MAGEE | CT10 | Xq27 | 1 | N/A | RDA | [10] |
| SPANX | CT11 | Xq27.1 | 4 | humoral (SPANXB1/CT11.2) | N/A | [11] |
| XAGE | CT12 | Xp11.22-p11.21 | 8 | N/A | Database mining | [12] |
| HAGE | CT13 | 6q12-q13 | 1 | N/A | RDA | [13] |
| SAGE | CT14 | Xq26 | 1 | N/A | RDA | [13] |
| ADAM2 | CT15 | 8p11.2 | 1 | N/A | Database mining | [14] |
| PAGE-5 | CT16 | Xp11.22 | 2 |  |  |  |
| LIPI | CT17 | 21q11.2 | 1 |  |  |  |
| NA88A pseudogene | CT18 | N/A | 1 | cellular | T cell epitope cloning | [15] |
| IL13RA1* | CT19 | Xq24 | 1 | N/A | ---- | [16] |
| TSP50* | CT20 | 3p14-p12 | 1 | N/A | ---- | [17] |
| CTAGE-1 | CT21 | 18p11.2 | 2 | humoral | SEREX | [18] |
| SPA17* | CT22 | 11q24.2 | 1 | humoral | ---- | [19] |
| OY-TES-1* | CT23 | 12p12-p13 | 1 | humoral | SEREX | [20] |
| CSAGE | CT24 | Xq28 | 2 | N/A | Differential display | [21] |
| MMA1 | CT25 | 21q22.2 | 2 | N/A | Oligonucleotide array analysis | [22] |
| CAGE | CT26 | Xp22 | 1 | humoral | SEREX | [23] |
| BORIS | CT27 | 20q13.31 | 1 | N/A | Homology searches for  paralogues to CTCF | [24] |
| HOM-TES-85 | CT28 | Xq24 | 1 | humoral | SEREX | [25] |
| D40/AF15q | CT29 | 15q14 | 1 | N/A | Yeast 2 hybrid screening | [26] |
| HCA661 | CT30 | Xq26.2 | 1 | humoral | SEREX | [27] |
| PLU-1* | CT31 | 1q32.1 | 1 | N/A | screening of the breast  cancer cDNA library | [28] |
| LDHC | CT32 | 11p15.5-p15.3 | 1 | N/A | Database mining | [29] |
| MORC | CT33 | 3q13 | 1 | N/A |  |  |
| SGY-1 | CT34 | 19q13.33 | 1 | N/A |  |  |
| SPO11 | CT35 | 20q13.2-q13.3 | 1 | N/A |  |  |
| TPX1 | CT36 | 9q12 | 1 | N/A |  |  |
| NY-SAR-35 | CT37 | Xq28 | 1 | humoral | SEREX | [30] |
| FTHL17 | CT38 | Xp21 | 1 | N/A | Search for orthologues  of murine testis-specific gene products | [31] |
| NXF2 | CT39 | Xq22.1 | 1 | N/A |  |  |
| TAF7L | CT40 | Xq22.1 | 1 | N/A |  | [32] |
| TDRD1 | CT41 | 10q26.11 | 2 | N/A |  | [31] |
| TEX15 | CT42 | 8p12 | 1 | N/A |  |  |
| FATE | CT43 | Xq28 | 1 | humoral | Database mining | [33] |
| TPTE | CT44 | 21p11 | 1 | humoral | Database mining | [33] |

*Ubiquitously expressed CT genes;

**CT nomenclature suggested by Scanlan et al. [34].

Compiled using data from the CT database (<http://www.cancerimmunity.org/ctdatabase>) and Locus Link (http://www.ncbi.nlm.nih.gov/LocusLink/).

References

1. van der BP, Traversari C, Chomez P, Lurquin C, De Plaen E, Van den EB, Knuth A, Boon T: **A gene encoding an antigen recognized by cytolytic T lymphocytes on a human melanoma.** *Science* 1991, **254:**1643-1647.

2. Boel P, Wildmann C, Sensi ML, Brasseur R, Renauld JC, Coulie P, Boon T, van der BP: **BAGE: a new gene encoding an antigen recognized on human melanomas by cytolytic T lymphocytes.** *Immunity* 1995, **2:**167-175.

3. Muscatelli F, Walker AP, De Plaen E, Stafford AN, Monaco AP: **Isolation and characterization of a MAGE gene family in the Xp21.3 region.** *Proc Natl Acad Sci U S A* 1995, **92:**4987-4991.

4. Van den EB, Peeters O, De Backer O, Gaugler B, Lucas S, Boon T: **A new family of genes coding for an antigen recognized by autologous cytolytic T lymphocytes on a human melanoma.** *J Exp Med* 1995, **182:**689-698.

5. Gure AO, Tureci O, Sahin U, Tsang S, Scanlan MJ, Jager E, Knuth A, Pfreundschuh M, Old LJ, Chen YT: **SSX: a multigene family with several members transcribed in normal testis and human cancer.** *Int J Cancer* 1997, **72:**965-971.

6. Chen YT, Scanlan MJ, Sahin U, Tureci O, Gure AO, Tsang S, Williamson B, Stockert E, Pfreundschuh M, Old LJ: **A testicular antigen aberrantly expressed in human cancers detected by autologous antibody screening.** *Proc Natl Acad Sci U S A* 1997, **94:**1914-1918.

7. Lucas S, De Smet C, Arden KC, Viars CS, Lethe B, Lurquin C, Boon T: **Identification of a new MAGE gene with tumor-specific expression by representational difference analysis.** *Cancer Res* 1998, **58:**743-752.

8. Tureci O, Sahin U, Zwick C, Koslowski M, Seitz G, Pfreundschuh M: **Identification of a meiosis-specific protein as a member of the class of cancer/testis antigens.** *Proc Natl Acad Sci U S A* 1998, **95:**5211-5216.

9. Scanlan MJ, Altorki NK, Gure AO, Williamson B, Jungbluth A, Chen YT, Old LJ: **Expression of cancer-testis antigens in lung cancer: definition of bromodomain testis-specific gene (BRDT) as a new CT gene, CT9.** *Cancer Lett* 2000, **150:**155-164.

10. Gure AO, Stockert E, Arden KC, Boyer AD, Viars CS, Scanlan MJ, Old LJ, Chen YT: **CT10: a new cancer-testis (CT) antigen homologous to CT7 and the MAGE family, identified by representational-difference analysis.** *Int J Cancer* 2000, **85:**726-732.

11. Westbrook VA, Diekman AB, Klotz KL, Khole VV, Kap-Herr C, Golden WL, Eddy RL, Shows TB, Stoler MH, Lee CY et al.: **Spermatid-specific expression of the novel X-linked gene product SPAN-X localized to the nucleus of human spermatozoa.** *Biol Reprod* 2000, **63:**469-481.

12. Liu XF, Helman LJ, Yeung C, Bera TK, Lee B, Pastan I: **XAGE-1, a new gene that is frequently expressed in Ewing's sarcoma.** *Cancer Res* 2000, **60:**4752-4755.

13. Martelange V, De Smet C, De Plaen E, Lurquin C, Boon T: **Identification on a human sarcoma of two new genes with tumor-specific expression.** *Cancer Res* 2000, **60:**3848-3855.

14. Scanlan MJ, Gordon CM, Williamson B, Lee SY, Chen YT, Stockert E, Jungbluth A, Ritter G, Jager D, Jager E et al.: **Identification of cancer/testis genes by database mining and mRNA expression analysis.** *Int J Cancer* 2002, **98:**485-492.

15. Moreau-Aubry A, Le Guiner S, Labarriere N, Gesnel MC, Jotereau F, Breathnach R: **A processed pseudogene codes for a new antigen recognized by a CD8(+) T cell clone on melanoma.** *J Exp Med* 2000, **191:**1617-1624.

16. Aman MJ, Tayebi N, Obiri NI, Puri RK, Modi WS, Leonard WJ: **cDNA cloning and characterization of the human interleukin 13 receptor alpha chain.** *J Biol Chem* 1996, **271:**29265-29270.

17. Yuan L, Shan J, De Risi D, Broome J, Lovecchio J, Gal D, Vinciguerra V, Xu HP: **Isolation of a novel gene, TSP50, by a hypomethylated DNA fragment in human breast cancer.** *Cancer Res* 1999, **59:**3215-3221.

18. Eichmuller S, Usener D, Dummer R, Stein A, Thiel D, Schadendorf D: **Serological detection of cutaneous T-cell lymphoma-associated antigens.** *Proc Natl Acad Sci U S A* 2001, **98:**629-634.

19. De Jong A, Buchli R, Robbins D: **Characterization of sperm protein 17 in human somatic and neoplastic tissue.** *Cancer Lett* 2002, **186:**201-209.

20. Ono T, Kurashige T, Harada N, Noguchi Y, Saika T, Niikawa N, Aoe M, Nakamura S, Higashi T, Hiraki A et al.: **Identification of proacrosin binding protein sp32 precursor as a human cancer/testis antigen.** *Proc Natl Acad Sci U S A* 2001, **98:**3282-3287.

21. Lin C, Mak S, Meitner PA, Wolf JM, Bluman EM, Block JA, Terek RM: **Cancer/testis antigen CSAGE is concurrently expressed with MAGE in chondrosarcoma.** *Gene* 2002, **285:**269-278.

22. de Wit NJ, Weidle UH, Ruiter DJ, van Muijen GN: **Expression profiling of MMA-1a and splice variant MMA-1b: new cancer/testis antigens identified in human melanoma.** *Int J Cancer* 2002, **98:**547-553.

23. Cho B, Lim Y, Lee DY, Park SY, Lee H, Kim WH, Yang H, Bang YJ, Jeoung DI: **Identification and characterization of a novel cancer/testis antigen gene CAGE.** *Biochem Biophys Res Commun* 2002, **292:**715-726.

24. Loukinov DI, Pugacheva E, Vatolin S, Pack SD, Moon H, Chernukhin I, Mannan P, Larsson E, Kanduri C, Vostrov AA et al.: **BORIS, a novel male germ-line-specific protein associated with epigenetic reprogramming events, shares the same 11-zinc-finger domain with CTCF, the insulator protein involved in reading imprinting marks in the soma.** *Proc Natl Acad Sci U S A* 2002, **99:**6806-6811.

25. Tureci O, Sahin U, Koslowski M, Buss B, Bell C, Ballweber P, Zwick C, Eberle T, Zuber M, Villena-Heinsen C et al.: **A novel tumour associated leucine zipper protein targeting to sites of gene transcription and splicing.** *Oncogene* 2002, **21:**3879-3888.

26. Takimoto M, Wei G, Dosaka-Akita H, Mao P, Kondo S, Sakuragi N, Chiba I, Miura T, Itoh N, Sasao T et al.: **Frequent expression of new cancer/testis gene D40/AF15q14 in lung cancers of smokers.** *Br J Cancer* 2002, **86:**1757-1762.

27. Wang Y, Han KJ, Pang XW, Vaughan HA, Qu W, Dong XY, Peng JR, Zhao HT, Rui JA, Leng XS et al.: **Large scale identification of human hepatocellular carcinoma-associated antigens by autoantibodies.** *J Immunol* 2002, **169:**1102-1109.

28. Lu PJ, Sundquist K, Baeckstrom D, Poulsom R, Hanby A, Meier-Ewert S, Jones T, Mitchell M, Pitha-Rowe P, Freemont P et al.: **A novel gene (PLU-1) containing highly conserved putative DNA/chromatin binding motifs is specifically up-regulated in breast cancer.** *J Biol Chem* 1999, **274:**15633-15645.

29. Koslowski M, Tureci O, Bell C, Krause P, Lehr HA, Brunner J, Seitz G, Nestle FO, Huber C, Sahin U: **Multiple splice variants of lactate dehydrogenase C selectively expressed in human cancer.** *Cancer Res* 2002, **62:**6750-6755.

30. Lee SY, Obata Y, Yoshida M, Stockert E, Williamson B, Jungbluth AA, Chen YT, Old LJ, Scanlan MJ: **Immunomic analysis of human sarcoma.** *Proc Natl Acad Sci U S A* 2003, **100:**2651-2656.

31. Loriot A, Boon T, De Smet C: **Five new human cancer-germline genes identified among 12 genes expressed in spermatogonia.** *Int J Cancer* 2003, **105:**371-376.

32. Wang PJ, McCarrey JR, Yang F, Page DC: **An abundance of X-linked genes expressed in spermatogonia.** *Nat Genet* 2001, **27:**422-426.

33. Dong XY, Su YR, Qian XP, Yang XA, Pang XW, Wu HY, Chen WF: **Identification of two novel CT antigens and their capacity to elicit antibody response in hepatocellular carcinoma patients.** *Br J Cancer* 2003, **89:**291-297.

34. Scanlan MJ, Simpson AJ, Old LJ: **The cancer/testis genes: review, standardization, and commentary.** *Cancer Immun* 2004, **4:**1.
