# Supplementary material for: Cancer/testis antigens and gametogenesis: a review and "brain-storming" session
Source: Cancer Cell Int. 2005 Feb 16;5:4. doi: 10.1186/1475-2867-5-4 (PMC552320; doi:10.1186/1475-2867-5-4)
Supplement: Additional File 2 — " CTA Frequency (%) of expression in various tumour types". [file 1475-2867-5-4-S2.doc]

**Additional file 2.**

**CTA Frequency (%) of expression in various tumour types.**

| CTA gene family (member) | Frequency (%) of Expression in tumour Type | | | | | | | | | | | | | | | | | | | |
| --- | --- | --- | --- | --- | --- | --- | --- | --- | --- | --- | --- | --- | --- | --- | --- | --- | --- | --- | --- | --- |
|  | Bladder | Brain | Breast | Colon | Esophageal | Gastric | Thyroid^d^ | Head and neck | Liver | Leukemia/ lymphoma | Lung (NSCLC) | Melanoma | Ovarian | Pancreatic | Prostate | Seminoma^a^ | Nonseminomatous germ cell tumour^a^ | Renal | Sarcoma | References |
|  |  |  |  |  |  |  |  |  |  |  |  |  |  |  |  |  |  |  |  |  |
| MAGEA1/CT1.1 | 22 | - | 18 | 2 | 53 | 29 | 17 | 28 | 80 | 0 | 49 | 48 | 28 | - | 15 | 54 | 20 | 0 | 14 | [1] |
| BAGE1/CT2.1 | 15 | - | 10 | 0 | - | - | 9 | 8 | - | 0 | 4 | 26 | 15 | - | 0 | - | - | 0 | 6 | [1] |
| MAGEB1/CT3.1 | 0 | 0 | 17 | 0 | - | 0 | - | 0 | - | 0 | 14 | 22 | - | - | 0 | 100 | 20 | 0 | 9 | [2] |
| GAGE/CT4.1 | 12 | - | 9 | 0 | - | - | 13 | 19 | 38^b^ | 1 | 19 | 28 | 31 | 21^c^ | 10 | 54 | 0 | 0 | 25 | [1] |
| SSX2/CT5.2 | 44 | 6 | 7 | 12 | - | - | 4 | 35 | 9^b^ | 36 | 16 | 35 | - | 0^c^ | 40 | - | - | 5 | 50 | [3] |
| NY-ESO-1/CT6.1 | 80 | 0 | 30 | 0 | - | 0 | 65 | - | 29 | 0 | 17 | 34 | 25 | 0 | 25 | 46 | 0 | 9 | 0 | [4] |
| MAGEC1/CT7.1 | 44 | - | 30 | 10 | - | - | - | 36 | - | - | 33 | 70 | - | - | - | - | - | - | 60 | [5] |
| SYCP1/CT8 | - | 47 | 20 | 0 | - | 7 | - | - | 28^b^ | 0 | 7 | 14 | 0 | 48^c^ | 0 | 39 | 0 | 8 | 0 | [3] |
| BRDT/CT9 | 0 | - | 0 | 0 | 8 | - | - | 8 | - | - | 25 | 0 | - | - | - | - | - | 0 | - | [6] |
| MAGEE1/CT10 | 44 | - | 38 | 0 | - | - | - | 36 | - | - | 24 | 50 | - | - | - | - | - | - | 0 | [7] |
| SPANXC/CT11.3 | 9 | - | 25 | 22 | 0 | - | - | - | - | - | 33 | 70 | - | 0 | - | - | - | - | - | [8] |
| XAGE-1a/CT12-1a | - | - | - | - | - | - | - | - | - | - | - | 8 | - | - | - | - | - | - | 22 | [9] |
| HAGE/CT13 | 24 | 37 | 5 | 31 | 27 | - | - | - | 20 | 9 | 32 | 17 | - | - | 22 | - | - | 6 | 20 | [10] |
| SAGE/CT14 | 12 | 0 | 5 | 0 | 20 | - | - | 17 | - | 4 | 22 | 4 | - | - | 0 | - | - | 5 | 5 | [10] |
| ADAM2/CT15 | - | - | 0 | 0 | - | - | - | - | - | - | 0 | 0 | 0 | - | - | - | - | 12 | - | [11] |
| PAGE-5/CT16 | - | - | 5 | 11 | - | - | - | - | - | - | 39 | 22 | 0 | - | - | 0 | 0 | 44 | - | [11] |
| LIPI/CT17 | - | - | 5 | 0 | - | - | - | - | - | - | 0 | 0 | 0 | - | - | - | - | 25 | - | [11] |
| NA88A /CT18 | - | - | - | - | - | - | - | - | - | - | - | 11 | - | - | - | - | - | - | - | [12] |
| TSP50/CT20 | - | - | 28 | - | - | - | - | - | - | - | - | - | - | - | - | - | - | - | - | [13] |
| CTAGE-1/CT21.1 | - | - | - | - | - | - | - | - | - | 35 | - | - | - | - | - | - | - | - | - | [14] |
| SPA17/CT22 | - | - | - | - | - | - | - | - | - | 26 | - | - | - | - | - | - | - | - | - | [15] |
| OYTES1/CT23 | 28 | - | 40 | 15 | - | 0 | - | - | 40 | - | 20 | - | - | - | - | - | - | 0 | - | [16] |
| MMA1a/CT25.1a | - | - | 0 | 0 | 0 | - | - | - | - | - | 40 | 26 | - | 0 | - | - | - | - | 18 | [17] |
| CAGE/CT26 | - | - | - | - | - | 89 | - | - | - | - | 100 | - | - | - | - | 54 | 0 | - | - | [18] |
| HOM-TES-85/CT28 | - | 35 | 0 | 10 | - | - | - | - | 19 | - | 28 | 36 | 32 | 5^c^ | 0 | - | - | - | - | [19] |
| D40(AF15q)/CT29 | - | 20 | - | 13 | - | 0 | - | - | - | - | 41 | - | 36 | 27 | - | - | - | - | - | [20] |
| HCA661/CT30 | 0 | - | - | - | - | 0 | - | 0 | 29 | - | - | 20 | - | - | - | - | - | - | - | [21] |
| PLU-1/CT31 | - | - | 86 | - | - | - | - | - | - | - | - | - | - | - | - | - | - | - | - | [22] |
| LDHC/CT32 | - | - | 35 | 15 | - | - |  | - | - | - | 47 | 44 | 42 | - | 37 | - | - | 57 | - | [23] |
| MORC/CT33 | - | - | 0 | 0 | - | - | - | - | - | - | 18 | 18 | 14 | - | 0 | - | - | 0 | - | [23] |
| SGY-1/CT34 | - | - | 20 | 0 | - | - | - | - | - | - | 12 | 25 | 57 | - | 12 | - | - | 0 | - | [23] |
| SPO11/CT35 | - | - | 0 | 0 | - | - | - | - | - | - | 0 | 6 | 0 | - | 0 | - | - | 0 | - | [23] |
| TPX1/CT36 | - | - | 15 | 0 | - | - | - | - | - | - | - | 6 | 14 | - | 37 | - | - | 14 | - | [23] |
| NYSAR35/CT37 | 42 | - | 23 | 0 | 8 | - | - | - | - | - | 17 | 6 | 8 | - | - | - | - | 0 | 8 | [24] |
| FTHL17/CT38 | 22 | - | 14 | 0 | 0 | - | - | 10 | - | 0 | 25 | 0 | - | - | 0 | - | - | 0 | 0 | [25] |
| NXF2/CT39 | 19 | - | 0 | 11 | 12 | - | - | 5 | - | 0 | 15 | 55 | - | - | 14 | - | - | 0 | 27 | [25] |
| TAF7L/CT40 | 10 | - | 0 | 0 | 0 | - | - | 10 | - | 0 | 9 | 21 | - | - | 0 | - | - | 0 | 12 | [25] |
| TDRD1/CT41.1 | 28 | - | 37 | 0 | 10 | - | - | 22 | - | 5 | 5 | 0 | - | - | 38 | - | - | 0 | 0 | [25] |
| TEX15/CT42 | 21 | - | 0 | 0 | 20 | - | - | 11 | - | 0 | 21 | 27 | - | - | 12 | - | - | 33 | 28 | [25] |
| FATE/CT43 | - | - | - | 21 | - | 7 | - | - | 66 | - | 0 | - | - | - | - | - | - | - | - | [26] |
| TPTE/CT44 | - | - | - | 0 | - | 0 | - | - | 39 | - | 36 | - | - | - | - | - | - | - | - | [26] |

Updated and completed from [27].

^a^ Refference: [28].

^b^ Refference: [29].

^c^ Refference: [30].

^d^ Refference : [31].

References

1. Van den Eynde BJ, van der BP: **T cell defined tumor antigens.** *Curr Opin Immunol* 1997, **9:**684-693.

2. Lurquin C, De Smet C, Brasseur F, Muscatelli F, Martelange V, De Plaen E, Brasseur R, Monaco AP, Boon T: **Two members of the human MAGEB gene family located in Xp21.3 are expressed in tumors of various histological origins.** *Genomics* 1997, **46:**397-408.

3. Tureci O, Sahin U, Zwick C, Koslowski M, Seitz G, Pfreundschuh M: **Identification of a meiosis-specific protein as a member of the class of cancer/testis antigens.** *Proc Natl Acad Sci U S A* 1998, **95:**5211-5216.

4. Chen YT, Scanlan MJ, Sahin U, Tureci O, Gure AO, Tsang S, Williamson B, Stockert E, Pfreundschuh M, Old LJ: **A testicular antigen aberrantly expressed in human cancers detected by autologous antibody screening.** *Proc Natl Acad Sci U S A* 1997, **94:**1914-1918.

5. Chen YT, Gure AO, Tsang S, Stockert E, Jager E, Knuth A, Old LJ: **Identification of multiple cancer/testis antigens by allogeneic antibody screening of a melanoma cell line library.** *Proc Natl Acad Sci U S A* 1998, **95:**6919-6923.

6. Scanlan MJ, Altorki NK, Gure AO, Williamson B, Jungbluth A, Chen YT, Old LJ: **Expression of cancer-testis antigens in lung cancer: definition of bromodomain testis-specific gene (BRDT) as a new CT gene, CT9.** *Cancer Lett* 2000, **150:**155-164.

7. Gure AO, Stockert E, Arden KC, Boyer AD, Viars CS, Scanlan MJ, Old LJ, Chen YT: **CT10: a new cancer-testis (CT) antigen homologous to CT7 and the MAGE family, identified by representational-difference analysis.** *Int J Cancer* 2000, **85:**726-732.

8. Zendman AJ, Cornelissen IM, Weidle UH, Ruiter DJ, van Muijen GN: **CTp11, a novel member of the family of human cancer/testis antigens.** *Cancer Res* 1999, **59:**6223-6229.

9. Zendman AJ, Van Kraats AA, Weidle UH, Ruiter DJ, Van Muijen GN: **The XAGE family of cancer/testis-associated genes: alignment and expression profile in normal tissues, melanoma lesions and Ewing's sarcoma.** *Int J Cancer* 2002, **99:**361-369.

10. Martelange V, De Smet C, De Plaen E, Lurquin C, Boon T: **Identification on a human sarcoma of two new genes with tumor-specific expression.** *Cancer Res* 2000, **60:**3848-3855.

11. Scanlan MJ, Gordon CM, Williamson B, Lee SY, Chen YT, Stockert E, Jungbluth A, Ritter G, Jager D, Jager E et al.: **Identification of cancer/testis genes by database mining and mRNA expression analysis.** *Int J Cancer* 2002, **98:**485-492.

12. Moreau-Aubry A, Le Guiner S, Labarriere N, Gesnel MC, Jotereau F, Breathnach R: **A processed pseudogene codes for a new antigen recognized by a CD8(+) T cell clone on melanoma.** *J Exp Med* 2000, **191:**1617-1624.

13. Yuan L, Shan J, De Risi D, Broome J, Lovecchio J, Gal D, Vinciguerra V, Xu HP: **Isolation of a novel gene, TSP50, by a hypomethylated DNA fragment in human breast cancer.** *Cancer Res* 1999, **59:**3215-3221.

14. Eichmuller S, Usener D, Dummer R, Stein A, Thiel D, Schadendorf D: **Serological detection of cutaneous T-cell lymphoma-associated antigens.** *Proc Natl Acad Sci U S A* 2001, **98:**629-634.

15. Lim SH, Wang Z, Chiriva-Internati M, Xue Y: **Sperm protein 17 is a novel cancer-testis antigen in multiple myeloma.** *Blood* 2001, **97:**1508-1510.

16. Ono T, Kurashige T, Harada N, Noguchi Y, Saika T, Niikawa N, Aoe M, Nakamura S, Higashi T, Hiraki A et al.: **Identification of proacrosin binding protein sp32 precursor as a human cancer/testis antigen.** *Proc Natl Acad Sci U S A* 2001, **98:**3282-3287.

17. de Wit NJ, Weidle UH, Ruiter DJ, van Muijen GN: **Expression profiling of MMA-1a and splice variant MMA-1b: new cancer/testis antigens identified in human melanoma.** *Int J Cancer* 2002, **98:**547-553.

18. Cho B, Lim Y, Lee DY, Park SY, Lee H, Kim WH, Yang H, Bang YJ, Jeoung DI: **Identification and characterization of a novel cancer/testis antigen gene CAGE.** *Biochem Biophys Res Commun* 2002, **292:**715-726.

19. Tureci O, Sahin U, Koslowski M, Buss B, Bell C, Ballweber P, Zwick C, Eberle T, Zuber M, Villena-Heinsen C et al.: **A novel tumour associated leucine zipper protein targeting to sites of gene transcription and splicing.** *Oncogene* 2002, **21:**3879-3888.

20. Takimoto M, Wei G, Dosaka-Akita H, Mao P, Kondo S, Sakuragi N, Chiba I, Miura T, Itoh N, Sasao T et al.: **Frequent expression of new cancer/testis gene D40/AF15q14 in lung cancers of smokers.** *Br J Cancer* 2002, **86:**1757-1762.

21. Wang Y, Han KJ, Pang XW, Vaughan HA, Qu W, Dong XY, Peng JR, Zhao HT, Rui JA, Leng XS et al.: **Large scale identification of human hepatocellular carcinoma-associated antigens by autoantibodies.** *J Immunol* 2002, **169:**1102-1109.

22. Barrett A, Madsen B, Copier J, Lu PJ, Cooper L, Scibetta AG, Burchell J, Taylor-Papadimitriou J: **PLU-1 nuclear protein, which is upregulated in breast cancer, shows restricted expression in normal human adult tissues: a new cancer/testis antigen?** *Int J Cancer* 2002, **101:**581-588.

23. Koslowski M, Tureci O, Bell C, Krause P, Lehr HA, Brunner J, Seitz G, Nestle FO, Huber C, Sahin U: **Multiple splice variants of lactate dehydrogenase C selectively expressed in human cancer.** *Cancer Res* 2002, **62:**6750-6755.

24. Lee SY, Obata Y, Yoshida M, Stockert E, Williamson B, Jungbluth AA, Chen YT, Old LJ, Scanlan MJ: **Immunomic analysis of human sarcoma.** *Proc Natl Acad Sci U S A* 2003, **100:**2651-2656.

25. Loriot A, Boon T, De Smet C: **Five new human cancer-germline genes identified among 12 genes expressed in spermatogonia.** *Int J Cancer* 2003, **105:**371-376.

26. Dong XY, Su YR, Qian XP, Yang XA, Pang XW, Wu HY, Chen WF: **Identification of two novel CT antigens and their capacity to elicit antibody response in hepatocellular carcinoma patients.** *Br J Cancer* 2003, **89:**291-297.

27. Scanlan MJ, Simpson AJ, Old LJ: **The cancer/testis genes: review, standardization, and commentary.** *Cancer Immun* 2004, **4:**1.

28. Yuasa T, Okamoto K, Kawakami T, Mishina M, Ogawa O, Okada Y: **Expression patterns of cancer testis antigens in testicular germ cell tumors and adjacent testicular tissue.** *J Urol* 2001, **165:**1790-1794.

29. Luo G, Huang S, Xie X, Stockert E, Chen YT, Kubuschok B, Pfreundschuh M: **Expression of cancer-testis genes in human hepatocellular carcinomas.** *Cancer Immun* 2002, **2:**11.

30. Kubuschok B, Xie X, Jesnowski R, Preuss KD, Romeike BF, Neumann F, Regitz E, Pistorius G, Schilling M, Scheunemann P et al.: **Expression of cancer testis antigens in pancreatic carcinoma cell lines, pancreatic adenocarcinoma and chronic pancreatitis.** *Int J Cancer* 2004, **109:**568-575.

31. Maio M, Coral S, Sigalotti L, Elisei R, Romei C, Rossi G, Cortini E, Colizzi F, Fenzi G, Altomonte M et al.: **Analysis of cancer/testis antigens in sporadic medullary thyroid carcinoma: expression and humoral response to NY-ESO-1.** *J Clin Endocrinol Metab* 2003, **88:**748-754.
